# The acheulean handaxe: More like a bird's song than a beatles' tune?

**DOI:** 10.1002/evan.21467

**Published:** 2016-01-22

**Authors:** Raymond Corbey, Adam Jagich, Krist Vaesen, Mark Collard

**Keywords:** Acheulean handaxe, cultural transmission, social learning, genetic transmission

## Abstract

**The goal of this paper is to provoke debate about the nature of an iconic artifact—the Acheulean handaxe. Specifically, we want to initiate a conversation about whether or not they are cultural objects. The vast majority of archeologists assume that the behaviors involved in the production of handaxes were acquired by social learning and that handaxes are therefore cultural. We will argue that this assumption is not warranted on the basis of the available evidence and that an alternative hypothesis should be given serious consideration. This alternative hypothesis is that the form of Acheulean handaxes was at least partly under genetic control.**

Named after the site of Saint‐Acheul in France, where they were first identified, Acheulean handaxes are distinctive (Fig. 1). Indeed, they are so distinctive that they are probably the one artifact that all archeologists, whatever their period of interest, are capable of identifying. Acheulean handaxes were produced by the bifacial reduction of a block or large flake blank around a single, long axis. They have a cutting edge in the secant plane, and range in shape from lanceolate through ovate to orbiculate.

**Figure 1:**
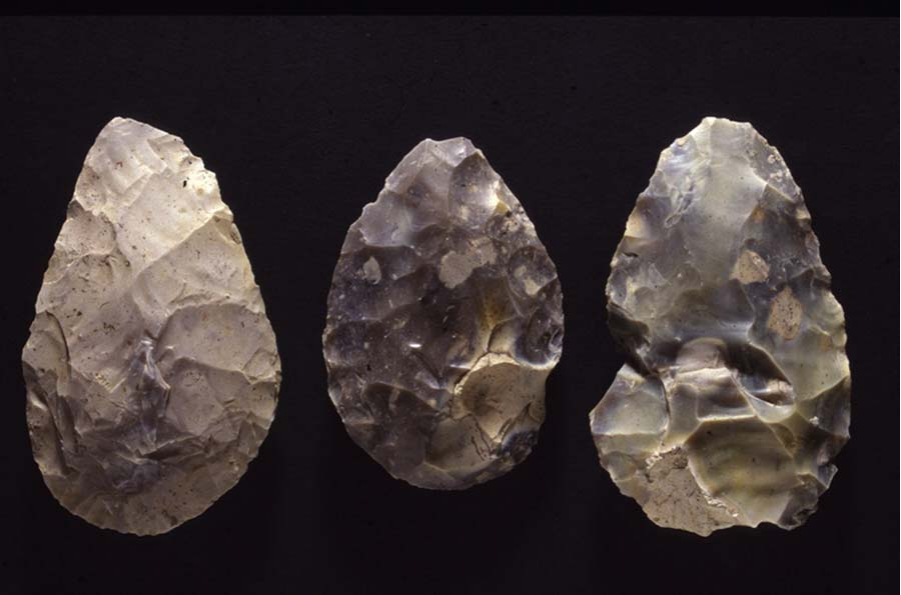
Acheulean handaxes from the site of Boxgrove, England, which dates to about 500 Ka. The handaxes are made of flint and are between 12 and 14.5 cm in length. Photograph by W. Roebroeks; used with permission. [Color figure can be viewed in the online issue, which is available at wileyonlinelibrary.com.]

Acheulean handaxes are one of the commonest, most widely distributed, and longest‐lasting archeological artifacts. Several hundred thousand Acheulean handaxes have been recovered from sites in many regions of the Old World, including North, South, and East Africa; Europe; and Western, South, and East Asia (Fig. 2). The oldest Acheulean handaxes date to approximately 1.76 million years ago (Ma)^1^ and the youngest to between 300 and 200 thousand years ago (Ka).^2^, ^3^ Thus, they span several million square kilometers, multiple ecological regions, and roughly a hundred thousand generations.

**Figure 2:**
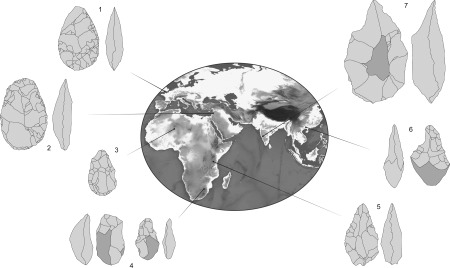
Acheulean handaxes from various regions (to scale; biface 7 is 22.5 cm tall). Sites: 1) Boxgrove, England; 2) North of Bridge Acheulean, near Gesher Benot Ya'aqov, Israel; 3) Erg Tihodaïne, Algeria; 4) Sterkfontein, South Africa; 5) Olduvai Gorge, Tanzania; 6) Bose, China, 7) Isampur, India.^89^, ^124^, ^125^, ^126^, ^127^, ^128^, ^129^ Figure by Shumon Hussain.

Acheulean handaxes are the defining artifact of the Acheulean industry, which also includes flakes, flake tools, and cores, as well as other large cutting tools such as cleavers, picks, trihedrals, and unifaces. The Acheulean industry was preceded by the Oldowan, which is found in Africa and parts of Eurasia; it was succeeded by the Middle Palaeolithic in western Eurasia and the Middle Stone Age in Africa.

Acheulean handaxes are thought to have been produced by two extinct hominin species, *Homo erectus* and *Homo heidelbergensis*. Fossils assigned to *H. erectus* have been recovered from sites in East Africa, South Africa, North Africa, the Caucasus, Southeast Asia, and East Asia.^5^ *H. erectus* is sometimes subdivided into *Homo ergaster* and *H. erectus sensu stricto*.^5^ The former is represented by several early African specimens and the latter by Eurasian and later African specimens. The hypodigm of *H. heidelbergensis* includes specimens from East Africa, South Africa, Europe, South Asia, and East Asia.^6^ *H. heidelbergensis* is sometimes argued to be a “wastebasket” taxon. Those who choose to split this taxon into two species usually assign Eurasian specimens to *H. heidelbergensis* and African specimens to *Homo rhodesiensis*.

Several issues regarding Acheulean handaxes are contentious. Most conspicuously, there is disagreement about their function. Most researchers consider handaxes to be cutting tools, but it has also been suggested that they were throwing weapons.^7^, ^8^, ^9^ In addition, it has been proposed that they played a role in social and/or sexual signaling.^10^, ^11^ There is also debate about the extent to which the form (that is, the shape and size) of Acheulean handaxes is deliberate. Most researchers assume that it is, but others assert that resharpening greatly affects handaxe form.^12^ According to this argument, resharpening generates similar forms in assemblages that are geographically and temporally separated. Still other researchers contend that raw material quality affected handaxe form,^13^ with, for example, large, flat chunks of flint from chalk cliffs yielding handaxes of a different form than small, elongated flint pebbles obtained from river beds.

There is even debate about the validity of the Acheulean handaxe as a type. The majority of researchers agree with McNabb^14^ that while handaxes may be large or small, more or less refined, pointed or ovate, symmetrical or off‐set, beneath this variability there are general “themes” that are present in all handaxes. However, there are some dissenting opinions. Nicoud,^15^ for instance, argues that the Acheulean handaxe is not a unitary phenomenon but rather an artificial grouping together of artifacts on the basis of superficial morphological and technological similarities.

Thus, disagreement about Acheulean handaxes abounds. However, as Richerson and Boyd^16^ observe, there is one thing that more or less all researchers working on handaxes agree on, which is that the behaviors necessary to manufacture them were copied from other individuals and, therefore, that handaxes are cultural objects. Richerson and Boyd^16^ offer an argument against this idea. They point out that both models and ethnographic data suggest that cultural learning in the small, relatively isolated groups that *H. erectus* and *H. heidelbergensis* are thought to have lived in should have resulted in rapidly diverging traditions rather than the “bewildering”^17^ ^:648^ geographic and temporal stability exhibited by the Acheulean handaxe. Based on this, Richerson and Boyd^16^ suggest that the conservatism of Acheulean handaxes may be evidence, not of cultural transmission, but of genetic transmission. Foley^18^ makes a similar point, arguing that since the pattern of handaxe variation does not match what we expect to see if the behaviors involved in their manufacture were socially learned, a role for genetic transmission should be considered.

To date, the possibility that the production of Acheulean handaxes was under at least partial genetic control has not been given serious consideration by archeologists. In our view, this is unfortunate. Here we attempt to explain why we think the genetic‐transmission hypothesis should be treated as a serious contender for explaining the handaxe phenomenon. We review several lines of evidence in favor of the hypothesis, as well as evidence against the dominant cultural transmission hypothesis. Subsequently, we use bird behaviors that have a substantial genetic component to illustrate how a behavior as complex as handaxe production could be under genetic control. In line with Richerson and Boyd,^16^ we will use the terms “social learning,” “cultural learning,” and “cultural transmission” interchangeably.

the possibility that the production of Acheulean handaxes was under at least partial genetic control has not been given serious consideration by archeologists. In our view, this is unfortunate. Here we attempt to explain why we think the genetic‐transmission hypothesis should be treated as a serious contender for explaining the handaxe phenomenon.

## THE DOMINANCE OF THE CULTURAL TRANSMISSION HYPOTHESIS

Until the 1990s, archeologists rarely discussed the manner in which the behaviors involved in the production of Acheulean handaxes were acquired. Handaxes were simply treated as cultural objects and the reader was left to infer that this meant the behaviors involved in their production were learned socially. In the 1990s, this began to change and, in line with developments elsewhere in the discipline of archeology, researchers working on Acheulean handaxes started to pay attention to the transmission of the behaviors involved in their production. Significantly, for present purposes, the transmission mechanisms suggested to be involved in the production of Acheulean handaxes in the vast majority of these publications, were cultural. We have located only a handful of publications, among them the aforementioned works by Boyd and Richerson^16^ and Foley,^18^ that suggest that something other than cultural transmission might have been involved in the acquisition of the behaviors required to produce handaxes.

Cultural transmission has been invoked numerous times in the post‐1990 literature to explain the stability of the key features of handaxes over roughly 1.5 million years. Mithen^19^ provides a clear example in his 1999 paper, “Imitation and cultural change: a view from the Stone Age, with specific reference to the manufacture of handaxes.” Observing the high degree of similarity in form among handaxes, Mithen argues that imitation must have been a “necessity for acquiring the technical skills to manufacture them.”^19:393^ Similarly, Shipton, Petraglia, and Paddayya^20^ state that imitation is crucial for understanding the conservatism of the Acheulean industry. They contend that “from the outset Acheulean social interactions were characterized by a propensity for true imitation and it is this, rather than any genetic predisposition to produce bifaces, which has produced the remarkable homogeneity of the Acheulean.”^20^ ^:229^ Shennan and Steele^21^ ^:368^ agree that imitative learning was crucial in the production of handaxes and even suggest that it was “most likely aided by active teaching.” Bar‐Yosef,^22^ in his concluding remarks to a widely cited edited volume on the Acheulean, supplies another example of the use of cultural transmission to explain the stability of the key features of handaxes. In a section entitled “The conservatism of the Acheulean knappers,” he argues that the retention of predetermined solutions was “dictated by cultural concepts.”^22:487^

In addition to being invoked to account for the stability of key features of the handaxe, cultural transmission has been used to explain the temporal variation in handaxe form. Over time, handaxes generally became smaller, thinner, and less elongated. They also became more standardized and more finely made.^23^ Furthermore, some studies have found that handaxes became more symmetrical as time progressed.^23^, ^24^, ^25^ Vaughan^4^ and Lycett^23^ used models from the field of population genetics to account for these changes. Despite borrowing their models from genetics, both authors frame their studies in terms of cultural evolution, and therefore implicitly assume that the behaviors involved in the manufacture of handaxes were socially learned.

Other authors have used cultural transmission to explain inter‐ and intra‐regional differences in handaxes. Mithen,^26^ ^:14^ for example, contends that “[t]here are clear, well defined handaxe types within the Acheulian, indicating cultural traditions in artefact form.”^1^ Later in the same paper he suggests that intersite differences may relate to “variability in the degree of experience and opportunities for social learning by individuals within and between hominid groups owing to variation in social interaction and group size.”^26^ ^:17^ Wynn and Tierson,^27^ Roe,^28^ and Wenban‐Smith^29^ make similar arguments. Wynn and Tierson^27^ hypothesize that variability among handaxes from Indian, British, Israeli, and African sites reflects regional cultural traditions, while Roe^28^ and Wenban‐Smith^29^ both posit cultural transmission of handaxe traditions within the British Palaeolithic.

The strength of the belief that Acheulean handaxes are cultural objects is underscored by a recent paper by Machin.^30^ She criticizes the standard approach to understanding handaxe variability, in which a single factor is viewed as being of primary importance. Instead, she contends, we need to recognize that multiple factors would have been involved. She goes on to outline no fewer than 48 factors that she believes were likely to have influenced handaxe variability. Significantly, for present purposes, at no point does she question the assumption that the behaviors required to produce handaxes were acquired by cultural transmission. The fact that even an author who calls on colleagues to think much more broadly about the way in which they approach handaxes does not question the assumption that handaxes are cultural objects shows just how deeply embedded the assumption is in archeology.

## REASONS TO DOUBT THE CULTURAL TRANSMISSION HYPOTHESIS

Earlier we explained why Richerson and Boyd^16^ and Foley^18^ suggest that a role for genetic transmission in the manufacture of handaxes should be considered. To reiterate, they point out that the long‐term conservatism of the Acheulean handaxe is inconsistent with models of cultural evolution and ethnographic data, which suggest that cultural transmission in groups of the type *H. erectus* and *H. heidelbergensis* are thought to have lived in should give rise to considerable spatiotemporal variability. That handaxes do not exhibit the kind of signal predicted by cultural evolutionary models and ethnographic data, Richerson and Boyd^16^ and Foley^18^ aver, implies that handaxes are not fully cultural objects, which in turn suggests that the behaviors involved in their manufacture may have been acquired at least in part by genetic transmission.

The most obvious objection to Richerson and Boyd's^16^ and Foley's^18^ argument is that it ignores the possibility that the form of handaxes was stable because it was strongly tied to a specific function. However, this rebuttal does not take into account copying error, which, because of the limitations of perception, is inevitable when learning how to make artifacts by copying another individual. Experiments involving human subjects indicate that copies typically deviate from their target by 3%‐5%, and that this error compounds over multiple transmission events.^31^ Over the course of almost 1.5 million years, 3%‐5% copying errors would have resulted in a substantial amount of spatiotemporal variation, regardless of how closely linked handaxes were to a particular function. This is not just a theoretical argument. A recent study found that size variation in a large sample of Acheulean handaxes was lower than expected from copying error.^32^ Given that copying error would inevitably have occurred if individuals had acquired the behaviors required to produce handaxes via social learning, this supports the idea that handaxes were not fully cultural artifacts and may have been under at least partial genetic control.

As strong as we think Richerson and Boyd's^16^ and Foley's^18^ argument is, we are of the opinion that it is just one of the problems facing the cultural transmission hypothesis. We will outline several other reasons to question the assumption that the behaviors involved in the manufacture of handaxes were acquired purely by cultural learning and to take seriously the possibility that they were acquired at least in part by genetic transmission. We believe that, taken together, Richerson and Boyd's^16^ and Foley's^18^ argument and the evidence we discuss in this section represent a powerful case against continuation of the current, uncritical approach to the nature of the Acheulean handaxe.

### The Chimpanzee Culture Debate Demonstrates That Mechanisms of Transmission Need to be Investigated and Not Simply Assumed

Another reason to question the assumption that Acheulean handaxes are cultural objects is provided by the ongoing debate about the nature of the behavioral differences among wild‐living chimpanzee groups. Over the last 40 years, numerous behaviors have been found to vary among chimpanzee groups. Some behaviors occur at some long‐term study sites but not at others. For instance, at the site of Bossou, Guinea, individuals often detach fronds from an oil palm and use them to smash the tree's crown to produce a pulp that is edible.^33^ So far, this behavior has not been documented at any other site.^34^ In addition, the way in which certain behaviors are performed varies among sites. Nut cracking illustrates this. At Bossou, only stone hammers and anvils are used to perform this task,^35^ whereas in the Taï Forest, Côte d'Ivoire, two different kinds of hammer, wood and stone, and two different kinds of anvil, root and stone, are used to crack nuts.^36^ An intersite comparison carried out a few years ago suggested that at least 65 behaviors vary among chimpanzee groups.^37^, ^38^

The nature of the among‐group behavioral differences is contested. According to some researchers, many of the behaviors are likely to be socially learned and therefore meet the main condition that is necessary for being recognized as cultural behaviors.^34^, ^35^, ^36^, ^37^, ^38^, ^39^, ^40^, ^41^ Other researchers question whether the behaviors are socially learned^42^, ^43^, ^44^, ^45^, ^46^ and highlight the fact that many of the putative cultural behaviors occur in a single subspecies. They point out that genetic studies suggest that some chimpanzee subspecies have been isolated for hundreds of thousands of years and contend that in these circumstances it is not possible to discount a genetic origin for the behavioral differences.

Currently, it is unclear which of these hypotheses is correct. Both have been supported by recent work. Lycett, Collard, and McGrew^47^, ^48^, ^49^ have reported several studies in which they compared phylogenetic trees derived from chimpanzee behavioral datasets to published genetic data pertaining to the relationships among chimpanzee subspecies and found that the behavioral trees did not match the genetic data. Based on this, these authors^47^, ^48^, ^49^ conclude that the culture hypothesis is more likely to be correct than is the genetic one. Langergraber and colleagues^46^ have reached a different conclusion. Using the Mantel test, they found a significant correlation between genetic and behavioral data for several chimpanzee groups. Based on this they argue that it is not possible to rule out a major role for genes in generating the differences among the groups' behavioral repertoires. There is disagreement about why the results of these studies do not converge,^50^, ^51^ which means that, at the moment, it is not possible to state with confidence that the behavioral differences among chimpanzee groups are cultural or genetic.

Thus, the nature of the behavioral differences among chimpanzee groups remains unclear even after nearly 40 years of discussion and completion of several research projects specifically designed to resolve the issue. This clearly indicates that it is not straightforward to infer mechanisms of transmission from behavioral patterns. An obvious corollary of this is that we should be skeptical when a mechanism of transmission has been concluded to be responsible for a behavioral pattern without any attempt to consider alternative mechanisms of transmission. This is the case with the Acheulean handaxe. Most researchers have simply assumed that the behaviors involved in the manufacture of handaxes were acquired by cultural transmission. As far as we are aware, no attempt has ever been made to determine whether the available data support the cultural transmission hypothesis better than they support potentially competing hypotheses such as individual learning and genetic transmission. Accordingly, the basis for believing that handaxes are cultural objects is shaky.

Most researchers have simply assumed that the behaviors involved in the manufacture of handaxes were acquired by cultural transmission. As far as we are aware, no attempt has ever been made to determine whether the available data support the cultural‐ transmission hypothesis better than they support potentially competing hypotheses such as individual learning and genetic transmission.

### Work on Modern Humans Also Indicates That Mechanisms of Transmission Need To Be Investigated and Not Simply Assumed

Recent research on living humans also suggests that we should be skeptical when a mechanism of transmission has been assumed to be responsible for a behavioral pattern without any attempt to consider alternative mechanisms of transmission. In 2004, McElreath^52^ reported a ground‐breaking study in which he sought to identify the processes underlying differences in the beliefs and behavior of farmers and pastoralists living in the Usangu Plains of Tanzania. He focused on individuals who had changed household economies, and investigated whether their dispositions with respect to three domains — preference for friends or kin, respect for elders, and belief in witchcraft — were a consequence of individual or social learning. McElreath's^52^ results were mixed. He found that variation in the prevalence of belief in witchcraft was best explained by social learning. However, variation in the other two domains was better explained by individual learning. Variation in whether or not individuals believed that respect for elders is important was strongly influenced by individual learning, but there was some effect of social learning. Variation in preference for friends or kin was not influenced at all by social learning, but only by individual learning. The main significance of these results for the present argument is that they demonstrate that even the beliefs and behaviors of modern humans cannot be simply assumed to be cultural phenomena. Many of them probably are, but some of them likely are not. As such, analysis is required before a given belief or behavior is deemed cultural.

To put it another way, McElreath's^52^ findings call into question the anthropological axiom that all the interesting beliefs and behaviors of modern humans are cultural. By extension, they also challenge the default assumption of paleoanthropologists that the behaviors of extinct hominins that are reflected in the archeological record, including the behaviors that resulted in Acheulean handaxes, are cultural. Many of the behaviors of modern humans likely are cultural, and many of the behaviors of extinct hominins probably were cultural. But it is not defensible to simply assume that this is the case. Rather, it needs to be demonstrated analytically. We need to treat the processes underlying the behaviors that anthropologists and archeologists study as unknown and design research projects to test between the competing hypotheses, in the same way that those working on the processes underlying behavioral variation among chimpanzee groups have tested between the culture hypothesis and the genetic hypothesis. Given the difficulty of associating artifacts with hominin species and the fragility of DNA, doing so will be difficult. However, this is not a good reason to ignore the problem.

### The Failure of Acheulean Handaxes To Track Environmental Variation Is Inconsistent with Models of Cultural Evolution

As we have seen, the long‐term conservatism of the Acheulean handaxe is inconsistent with a key prediction of recent models of cultural evolution, namely that cultural entities should exhibit considerable spatiotemporal variability. Handaxes are inconsistent with models of cultural evolution in other ways.

Modeling work carried out over the last 30 years or so sets fairly stringent conditions on the emergence of cultural transmission.^53^, ^54^ The goal of this work was to determine when cultural learning is favored over individual learning and genetic transmission, given that cultural learning is cognitively costly. For present purposes, the key finding is that cultural learning evolves only in moderately variable environments. In slowly changing environments, genetic transmission outperforms cultural transmission, while in highly variable environments individual learning beats both genetic transmission and cultural transmission. Equally importantly, in order for cultural transmission to be favored over the alternatives it must result in behaviors that track environmental conditions. If it does not give rise to such behaviors, genetic transmission or individual learning will be preferred by selection, depending on the speed of environmental change.

For the behavioral routines involved in the production of handaxes to have been culturally transmitted, therefore, the environments in which handaxes were produced must have been variable and handaxes must have tracked that variability. The first condition is met. It is clear that Acheulean toolmakers had to cope with fluctuating climates.^55^, ^56^, ^57^, ^58^ They also expanded into various new habitats.^55^, ^56^ Thus, there is reason to think that the environments in which handaxes were produced were variable. In contrast, the second condition is not met. As we explained earlier, the key features of the handaxe do not vary much across space or through time. The implication of this is that handaxes did not respond to the environmental variability that Acheulean toolmakers experienced. Given that cultural evolutionary models suggest the ability to track environmental variability is the reason why cultural transmission is sometimes favored over genetic transmission, the fact that handaxes do not track environmental variability presents another substantial challenge to the cultural transmission hypothesis.

### Explaining the Increased Speed of Cultural Change in The Late Pleistocene Is Difficult If Acheulean Handaxes Are Assumed To Be Fully Cultural

A further problem facing the cultural transmission hypothesis is the much more rapid pace of change after Acheulean handaxes disappeared at 300‐200 Ka. If the behaviors involved in the production of both handaxes and post‐Acheulean artifacts were culturally learned, how do we explain this very marked increase in the speed of change? Currently, two hypotheses prevail. Unfortunately, neither is particularly convincing.

The first hypothesis focuses on cognition. According to this hypothesis, the shift in speed occurred because the hominin brain was “upgraded”; that is, one or more cognitive capacities, such as enhanced working memory, causal reasoning, or executive control, were added.^59^, ^60^, ^61^, ^62^, ^63^ These heritable biological changes introduced the necessary cultural variation, so that the cultural evolutionary process could yield more complex culture. This scenario faces several problems, depending on when one sets the date of the event. If the event is set around 100 Ka, one can account for the sporadic appearance of many markers of modern behavior at multiple African sites as early as 70 to 90 ka. However, one cannot explain the disappearance of these markers between 75‐60 Ka and their later reemergence at different times in various parts of the world. If the event is set later, say right before the transition in Europe (45 Ka), one needs to assume that the arrival of fully modern behavior in southeast Asia and Australia resulted from either convergent evolution or dispersal out of Europe. The former seems unlikely, given the drastic nature of the change, while the latter is hard to reconcile with the available evidence on Late Pleistocene migrations. Furthermore, unless convergent evolution is invoked, increased cognitive ability would be denied to Neanderthals, despite compelling arguments to the contrary.^64^, ^65^, ^66^

The second potential explanation for the increase in speed of artifact change at 300–200 ka concentrates on demography. Hominin population size is thought to have increased in the Late Pleistocene and it has been argued that this would have given rise to an increase in cultural complexity. The main intuition behind the dependence of cultural change on demography is that larger populations can sustain a more complex culture because they are more likely to contain individuals whose skill level is at least as good as that of the individuals in the previous generation. However, the demographic explanation of the transition toward fully modern human behavior is problematic in at least two respects. First, it has been shown that the formal model that is used most frequently to support the demography hypothesis^67^, ^68^ only predicts a relationship between population size and cultural complexity in implausible conditions.^69^, ^70^ Second, a key prediction of the hypothesis — that there should be a significant, positive relationship between population size and cultural complexity in hunter‐gatherers — is not supported empirically. Several studies have found no evidence of an impact of population size on technological richness and complexity in ethnographically documented hunter‐gatherers.^71^, ^72^, ^73^, ^74^ A similar finding was obtained in a recent attempt to use North American archeological data to evaluate the impact of population size on hunter‐gatherer technology.^75^ Moreover, results of attempts to estimate the population sizes of Late Pleistocene human populations conflict with the timing of the transition toward fully modern human behavior.^76^

Given that neither of the cultural hypotheses withstands scrutiny, how do we explain the shift in speed of artifact change 300‐200 ka? If Acheulean handaxe production behavior was under genetic control, the post‐Acheulean speeding up and diversification of culture perhaps marked another event, namely greater reliance on cultural inheritance. This, in turn, may imply either the evolution of special‐purpose mechanisms for cultural learning or domain‐general mechanisms for learning being “tuned,” ontogenetically, to inputs from other agents.^77^

The first option seems to suffer from the same problems as those noted in relation to the cognitive upgrade hypothesis. The second option is more promising because it leaves room for cultural differences within *Homo sapiens* (geographically and diachronically) and between *H. sapiens* and Neanderthals, as a result of differences in ontogeny. According to this hypothesis, the tempo of cultural evolution depends on the extent to which attentional, perceptual, and/or motivational mechanisms are ontogenetically biased toward others. The Late Pleistocene transition may be a consequence of hominins starting to rely on cultural learning for a wide range of behaviors, due, for instance, to local conditions favoring increased social tolerance and/or the emergence of new types of social organization in which contact between putative pupils and models is more likely.

With regard to the latter possibility, a recent study by Hill and coworkers^78^ indicates that it is common for contemporary hunter‐gatherers to live in residential units with a high percentage of unrelated individuals. Such organization implies friendly visits between units and many more opportunities for observing novel cultural traits than is the case when residential units are primarily kin‐based. The emergence of these sorts of networks may thus be one of the ways in which human learning became more attuned to social cues. Also, if group members are not closely related, one should expect a variety of mechanisms that actively promote cooperation (indirect reciprocity, sanctioning of defectors). Under these conditions, it seems likely that social stimuli provide reliable information, which, according to Heyes,^77^ in turn favors higher attentiveness to such stimuli.

Religious beliefs and practices, which have been suggested to promote intragroup cohesiveness and cooperation.^79^, ^80^, ^81^, ^82^ may be another factor facilitating the ontogenetic development of cultural learning. In collective rituals, groups express and reaffirm shared beliefs, norms, and values, and this enhances communal stability and group harmony.^83^, ^84^ In addition, belief in supernatural entities that monitor their devotees and punish them in case of transgressions has been proposed to encourage prosociality towards strangers.^85^, ^86^ Religion would thus seem to provide suitable conditions for cultural learning and therefore allow the formation of cumulative culture.

To summarize, the increase in the pace of artifact change after the Acheulean may not be the consequence of a cognitive upgrade or increased population size, as archeologists often suppose. Instead, it may indicate a shift from genetic transmission to increasing reliance on ontogenetic cultural learning. This proposal offers a plausible mechanism by which artifact change could accelerate. It also is easier to reconcile with geographic and temporal variation than is the cultural hypothesis.

### Some Geographic Facts About Handaxes Are More Consistent With Genetic Transmission Than With Cultural Transmission

In the late 1940s, Movius^87^, ^88^ drew attention to the geographic distribution of Acheulean handaxes. He pointed out that handaxes had been found in Africa, Europe, and India, but not in East and Southeast Asia. This “Movius Line” persisted for many years, but recently handaxes have been found at various sites in the Bose Basin and other river basins in China, some 1,500 km northeast of the Movius Line.^89^ This unexpected development presents a third major problem for the cultural transmission hypothesis.

The unexpected occurrence of handaxes in China has been explained in terms of raw material distribution. It has been suggested that high‐quality lithic raw materials are rare in the eastern part of the range of *H. erectus*, but forest fires periodically uncovered suitable raw material in river basins. Whenever this happened, so the hypothesis goes, the hominins occupying the area produced bifaces. On the face of it, this seems like a reasonable suggestion. However, it actually ignores a critically important question: How were the behaviors involved in the production of handaxes acquired by hominins located 1,500 km from the Movius Line?

There are two potential answers to this question that involve cultural transmission, neither of which is particularly compelling. One possibility is that the Chinese hominins maintained contact with groups that regularly produced handaxes far to the west. The other is that Chinese hominins transmitted the knowledge of handaxe manufacture across many generations without ever producing them. Given what little we know about population dynamics, life history traits, and patterns of distribution so deep in the past both scenarios seem unlikely, especially in view of the vulnerability of cultural transmission to information loss.^90^

In contrast, the genetic transmission hypothesis straightforwardly explains the occurrence of handaxes 1,500 km from the Movius line: The genes responsible for the maintenance of such behaviors remained unexpressed in the population until proper raw material became available, at which time the relevant genes were expressed. The feasibility of this scenario is illustrated by studies showing that organisms are able to respond to changing environmental conditions by alterations to gene expression.^91^

A fourth potential explanation for the Chinese handaxes is worth noting. It is possible that the handaxes are the result of convergent evolution; that is, hominins in China developed handaxes independently from hominins on the west side of the Movius Line, so that the Chinese handaxes are not, in fact, Acheulean handaxes. This is possible; over the last few decades, it has become clear that convergent evolution is commonplace.^92^, ^93^, ^94^ However, the notion that the Chinese handaxes and Acheulean handaxes were invented independently is a less parsimonious explanation than the gene expression‐based explanation.

There is another geographic fact about handaxes that is illuminating to consider from the perspective of the genetic transmission hypothesis, and that is the way in which variability in symmetry changes with distance from Africa. Using a model derived from population genetics, Lycett^23^ tested aspects of handaxe symmetry in relation to predictions derived from the neutral theory of evolution. His analyses indicated that the intra‐assemblage variability of handaxe symmetry decreases with distance from Africa and does so in a way that is inconsistent with iterative founder effect. On the basis of this, Lycett^23^ argues that aspects of handaxe symmetry were subject to selective forces. Not surprisingly, given the strength of belief in the field that handaxes are cultural objects, Lycett,^23^ throughout his paper, assumes that the behaviors involved in handaxe manufacture were culturally learned. Lycett mentions the possibility that genetics influenced the design of handaxes, but does not take it seriously. We respectfully suggest that this is a mistake. If the variation in handaxe symmetry fits population genetics models, it stands to reason that the behaviors involved in the production of handaxes may have been acquired by genetic transmission.

### Recent Evidence from Neuroscience Is Consistent with the Genetic Transmission Hypothesis

Neuroscience has shown that present‐day humans can gauge intentions and feelings and can copy and predict the actions of others via mirror neurons.^95^, ^96^ At first sight, this seems to support the current approach to Acheulean handaxes in terms of domain‐general social learning. However, studies have begun to link mirror neurons with domain‐specific genetic preprogramming. Del Giudice, Manera, and Keysers^97^ argue that it is “reasonable to expect that an ability as crucial for survival as action recognition and learning through observation would become pre‐programmed (‘innate’) to some degree during phylogenetic history.”^97^ ^:351^ They go on to review empirical evidence suggesting that Hebbian learning, in which simultaneous activation of neurons leads to increased interconnection between those neurons, and genetic preprogramming occur specifically in connection with manual action.

Another relevant insight from neuroscience is that the neuronal circuitry in the human brain responsible for language production not only overlaps considerably with areas controlling manipulation but also is under strong genetic control. Stout and colleagues^98^, ^99^ argue that both tool‐making and speech are multilevel, hierarchically nested, sequential, and goal‐directed motor sequences. They summarize their argument as follows: “The observed patterns of activation and of overlap with language circuits suggest that tool‐making and language share a basis in more general human capacities for complex, goal‐directed action. The results are consistent with co‐evolutionary hypotheses linking the emergence of language, tool‐making, population‐level functional lateralization and association cortex expansion in human evolution.”^99^ We think this line of enquiry is promising with respect to a possible genetic component in handaxe design, but suspect that the capacities in question may well turn out to be much more specific than Stout et al. suggest. Relevant points of comparison between tool‐making and speech are manual control and articulatory control, action sequences and syntax, as well as mental templates and semantics, with bird song as a promising third relevant field.^100^

## BIRD MODELS FOR HANDAXE MANUFACTURE

An obvious concern about the genetic transmission hypothesis is whether the production of an object as complex as an Acheulean handaxe could really be under genetic control. We contend that the idea is not implausible if handaxe production is compared to some complex bird behaviors. We begin by discussing bird song and bird tool‐use as “soft” options that involve a combination of genetic influence and social learning. We then discuss a “hard” option — structure building by birds, in which strong genetic determination interacts with the availability of raw material and local environmental conditions but not with much, if any, cultural learning.

### Bird Song and Tool‐Use

Bird song, a well‐studied behavior, is strongly influenced by genetic predispositions and implemented by discrete, well‐defined neural circuits.^101^ Songbirds, hummingbirds, and parrots have fixed, species‐wide basic songs from which individuals and regional subpopulations develop their own varieties. Auditory‐guided vocal motor learning is important, for when members of these taxa are raised in isolation their songs remain simple. The genetically controlled early song of zebra finches (*Taeniopygia guttata*), for instance, is plastic. The young need several months of social learning to perfect their individual, adult song.^102^

It appears that in some bird species, tool use also combines fixed species‐wide basic motor behaviors with cultural learning. It has been found, for example, that hand‐raised Caledonian crows (*Corvus moneduloides*) reliably develop leaf‐tool manufacture without ever having observed it in others, but never develop the sophisticated behaviors found in wild populations where individuals are exposed to models and competitors. A study by Kenward and coworkers^103^ illustrates this. By hand‐rearing several crows in isolation, they showed that the tool behavior of isolated individuals was so similar to that in wild ones that it was difficult to avoid the conclusion that the behavior is innate. They argue against “the extreme possibilities that tool‐use depends entirely on social inputs (i.e., is sustained exclusively by cultural transmission and thus does not reflect a dedicated evolved adaptation), and that it has a purely individual, insight‐based origin.”^103^ ^:1340^ If that were the case, Kenward and colleagues^103^ ^:1340^ contend, we would not see “inherited action patterns that must have evolved through selection and that are crucial in sustaining tool‐oriented behavior in adult crows.” The same point has been made with respect to the tool‐use behavior of hyacinth macaws, Egyptian vultures, and woodpecker finches, the only other bird species that is known to habitually use stick tools in the wild.^104^, ^105^, ^106^

Thus, if modeled on bird song and bird tool‐use, the production of Acheulean handaxes would have involved both genetic transmission and social learning. Raw material selection, the manufacturing process, and basic design principles would have been under genetic control, but fine‐tuned through social learning. The latter would have been dependent on the presence of role models during sensitive periods, but would not necessarily have involved cultural group‐specific templates or explicit instruction. The combination of genetic transmission and social learning is predicted to produce uniformity of overall design (due to the fixed component) and slight local variance (due to the process of socialization), which is the pattern observed with Acheulean handaxes.

### Structure Building by Bird*s*

It appears that one important reason why archeologists tend to think that the behaviors involved in the production of handaxes were socially learned is that the handaxe *chaine opératoire* required multiple decisions at multiple stages, from the selection of the size, shape, and quality of raw material to the final retouch.^15^ Again, this argument seems reasonable on its face. However, it is not as secure as it first appears. The reason for this is that the construction of nests and other structures by birds often involves long hierarchical sequences even though, as is generally agreed, the required behaviors do not involve much, if any, cultural learning.

**Figure 3:**
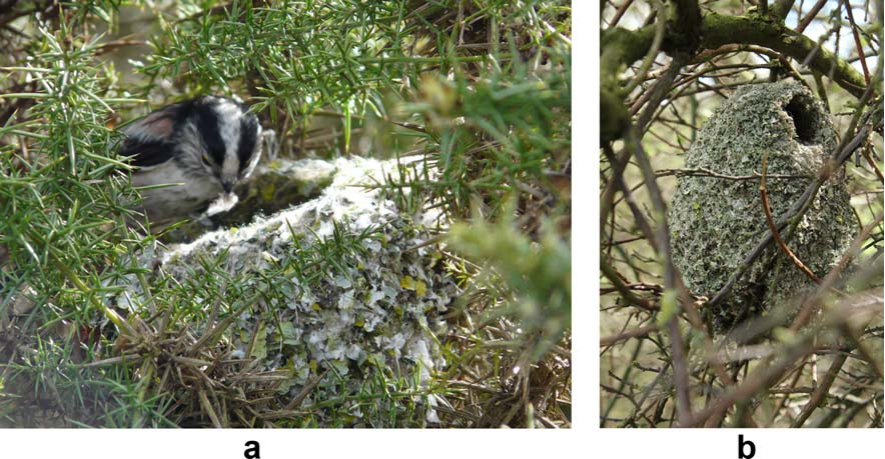
Nests created by long‐tailed tits (*Aegithalos caudatus*), Great Britain. Long‐ tailed tits build their nests from thousands of pieces of lichen, moss, and spiderweb, as well as numerous feathers. The nests are thought to be a result of innate instructions and individual learning. Photograph A shows a nest under construction, while photograph B shows a completed nest. (Photograph A: Alan Shearman/Wikimedia Commons; Photograph B: nottsexminer/Wikimedia Commons). [Color figure can be viewed in the online issue, which is available at wileyonlinelibrary.com.]

Eurasian long‐tailed tits (*Aegithalos caudatus*) exemplify the potential complexity of nest building behavior. These birds create intricate spherical nests from thousands of pieces of lichen, moss, and spiderweb, as well as numerous feathers (Fig. 3). The final product is accomplished through a combination of innate instructions and individual learning.^107^, ^108^ The former specifies materials and methods and a limited repertoire of repetitive, stereotyped actions. There is a chain of stimuli and responses that presupposes construction rules and local insight, but not necessarily complex, overall planning, conscious decision‐making, or a mental image of the ultimate goal. The nest is an emergent property of the stimulus‐response chain.

**Figure 4:**
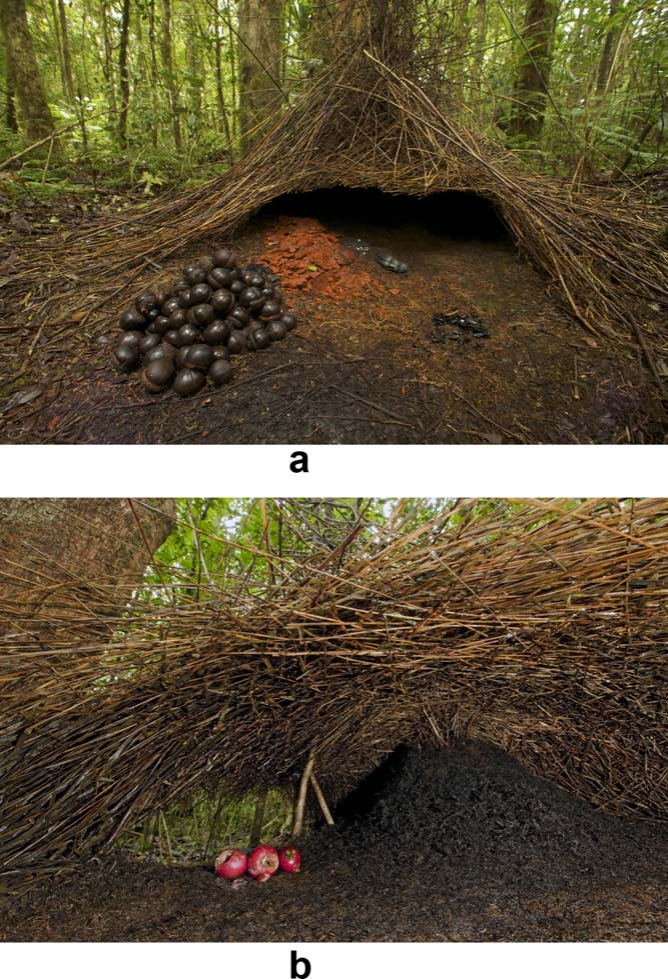
Bowers of the Vogelkop bowerbird (*Amblyornis inornatus*), western New Guinea. It is thought that the within‐species stability of the basic form of these bowers indicates that genes underlie their design and construction. Note the organization of colorful and shiny items in the area in front of the bower in photograph A. In photograph B, note the way in which the sticks that form the bower are intricately woven together. (Photograph A: Tim Laman/naturepl.com; photograph B: Ingo Arndt/naturepl.com). [Color figure can be viewed in the online issue, which is available at wileyonlinelibrary.com.]

The bower‐birds (Ptilonorhynchidae) of New Guinea provide another excellent set of examples. These birds create hut‐like shelters and decorate their interiors and the area immediately in front of them with brightly colored objects (Fig. 4). These “bowers” are individually and ecologically variable as a result of individual learning and, possibly, some cultural learning. It is thought, however, that the stability of their basic form indicates that genes underlie their design and construction.^109^

As with handaxes, the bowers show a mix of variation and uniformity. Individual bowers display variation, but share a general form within a species. The same can be said for handaxes: Beneath their variability, general themes are present in all of them.

Structure building by birds is also heuristically relevant when trying to clarify exactly what was under genetic control in handaxe manufacture. It probably was not just a simple target form, but rather a predisposition toward the basic behavioral routines involved, such as invasive bifacial reduction while realizing cutting edges in the secant plane, working from the tip down, and keeping symmetry. These routines would have operated in combination with causal understanding, manipulative skill, and intuitive (“folk”) physics.^110^ Although Gowlett^111^ ^:217^ thinks of handaxes as cultural objects, our proposal is consistent with his approach when he argues against the notion of a template as being “hard and fast” and instead talks of “instruction sets [from which] a computer could generate the form of a biface mindlessly.”

Unfortunately, genetic evolution usually happens too slowly for researchers to adequately study changes in genetically controlled behaviors such as nest building by birds. The time needed to observe changes in evolutionary trends extends far beyond the current length of study of such phenomena. Direct comparisons between the handaxe and animal behaviors such as birds' tool use or structure building are therefore difficult. Nevertheless, mounting evidence points to a central role for genes in maintaining aspects of animal technology. We suggest that the same should be considered for Acheulean handaxes.

## THE NATURE OF OTHER HANDAXE‐LIKE TOOLS

It is important, under the genetic transmission hypothesis, to clarify the nature of the other large cutting tools in the Acheulean industry and the handaxe‐like bifaces in the industries that come after the Acheulean. Were these tools also partly genetically determined or were they solely cultural phenomena?

McElreath's^52^ discovery that in his study‐groups different mechanisms underpinned attitudes toward friends versus kin, respect for elders, and belief in witchcraft clearly indicates that there is no reason to expect all Acheulean tools to involve the same transmission mechanisms. Nevertheless, we are of the opinion that the applicability of the genetic transmission hypothesis should be investigated in connection with the other large cutting tools of the Acheulean, among them cleavers, picks, trihedrals, and unifaces. Cleavers appear to be a particularly good candidate for another large cutting tool that was under at least partial genetic control. Cleavers are bifacially reduced pieces with a straight cutting edge perpendicular to the long axis of the piece. They were mostly made on flake blanks struck from bifacially prepared, usually more or less tortoise‐shaped cores. The cleaver has been argued to be a subcategory of the Acheulean handaxe. If this is the case, then parsimony suggests they could have been under partial genetic control as well.

We suspect the situation is different with respect to the handaxe‐like tools in the industries that came after the Acheulean. As we explained earlier, in Europe the Acheulean was replaced by the industries of the Middle Paleolithic between 300 and 200 Ka. Several European Late Middle Paleolithic industries contain bifacial tools, including the French Mousterian of Acheulean Tradition and the Middle European Keilmessergruppen, also called the Micoquian.^112^, ^113^ The tools in question resemble Acheulean handaxes, but we think it is unlikely that they were under genetic control.

There are two reasons for this. First, Late Middle Paleolithic bifaces are actually only superficially similar to Acheulean handaxes. A recent morphometric comparison of Acheulean and Late Middle Palaeolithic handaxes from Western Europe done by Iovita and McPherron^12^ ^:69^ demonstrated that Acheulean handaxes show an allometric pattern consistent with reduction of the tip, while Late Middle Palaeolithic handaxes “show a pattern which implies a maintenance of shape throughout the reduction continuum.” The authors conclude from this that the two tool types are unrelated. In addition, Late Middle Paleolithic bifaces seem to have been used in a different way than Acheulean handaxes. The latter were primarily platforms on which various working edges (for example, a point, a notch, or cutting edge) were made, whereas Middle Paleolithic bifaces usually served as complete tools with well integrated working edges that remained integrated during resharpening.^12^, ^112^, ^113^

The other reason we think it unlikely that Late Middle Paleolithic bifaces were under genetic control is that the industries they were part of were geographically restricted and relatively short lived. Ruebens^114^ has recently reported a typo‐technological analysis of bifacial tools from late Middle Palaeolithic sites in several European countries, including Britain, Belgium, the Netherlands, France and Germany. She found evidence of the existence of three different regional entities, the Mousterian of Acheulean Tradition, the Keilmessergruppen, and a new entity, which she calls “the Mousterian with Bifacial Tools.” The Mousterian of Acheulean Tradition, located in southwest Europe, is dominated by handaxes. The Keilmessergruppen is found in northeast Europe and is distinguished by backed and leaf‐shaped bifacial tools. The Mousterian with Bifacial Tools is located between the Mousterian of Acheulean Tradition and the Keilmessergruppen and contains a wider variety of bifacial tools than either of them. That these and other Late Middle Paleolithic industries were geographically restricted and short‐lived means they match what we expect to see if the behaviors involved in their manufacture were socially learned.^16^, ^18^ The corollary of this is that Late Middle Paleolithic bifaces are likely purely cultural objects.

Based on the results of their morphometric analysis, Iovita and McPherron^12^ argue that Acheulean handaxes and Middle Paleolithic bifaces reflect “different and likely unrelated cultural and human evolutionary contexts.” We suggest a different scenario in which genetic transmission stopped being a factor in the behavioral routines involved in the production of Middle Paleolithic bifaces. How and why this happened is an intriguing question but, given the connection between cultural learning and environmental variability discussed earlier, a link with an increase in environmental variability seems likely.^55^, ^56^, ^57^, ^58^

## CONCLUSIONS

The remarkably conservative character of the Acheulean handaxe — limited variability in key aspects of form over roughly 1.5 million years across two continents, in almost every type of ecology occupied by archaic hominins — is almost unanimously explained by cultural learning, variously combined with such factors as individual learning, functional requirements, raw material constraints, similar ecology, reuse and resharpening, and drift. In light of this apparent consensus, one would expect that the status of handaxes as cultural objects was settled through careful study a long time ago and is backed by a substantial body of evidence. This is not the case, however. The assumption that cultural transmission must have been the mechanism responsible for the conservation of specific design characteristic of the handaxe can be traced back to the early nineteenth century, when they were first discovered in France. In the absence of concepts like genes or instinct, handaxes were automatically perceived as cultural products, similar to those of that period's booming industrial technology. Crucially, the cultural transmission hypothesis has gone untested ever since. Handaxe production was, and still is, assumed by most archeologists to be underpinned by social learning. Therefore, handaxes are treated as cultural objects.

The cultural transmission hypothesis, however, faces several problems. It underestimates the difficulty of inferring transmission mechanisms from artifacts. It conflicts with cultural evolutionary models and ethnographic data on key characteristics of cultural objects. Moreover, this hypothesis is difficult to square with the greatly increased rate of technological change observed in post‐Acheulean industries. It also fails to account adequately for the handaxes that have recently been found beyond the Movius Line. We have argued that these and other problems can be better accommodated by what we call the genetic transmission hypothesis, which is that handaxe production was passed between generations at least in part through genetic inheritance. In addition, there are two strands of positive evidence for the genetic transmission hypothesis. First, handaxe variation accords with predictions from models of genetic evolution. Second, neuroscientific findings suggest that simple tool use may be the result of domain‐specific genetic preprogramming.

there are two strands of positive evidence for the genetic hypothesis. First, handaxe variation accords with predictions from models of genetic evolution; second, neuroscientific findings suggest that simple tool use may be the result of domain‐specific genetic preprogramming.

In this paper, we have adopted dual‐inheritance theory's^16^, ^53^, ^115^, ^116^ distinction between two channels of inheritance, genetic and cultural, even though, in reality, traits may very well resist such neat partitioning.^16^ We recognize that in doing so we have opened ourselves up to the charge of simply replacing one simplistic account with another simplistic implausible story. Even though this may turn out to be correct, we hope that setting up the issue in terms of a dichotomy will spark a debate on what has been unquestionably assumed by the vast majority of archeologists for more than a century.

It may be helpful to clarify what the genetic transmission hypothesis does and does not entail. First, it does not imply strong instructionism, which is the idea that genes code for handaxe production in the sense of being uniquely responsible for specifying that behavior.^117^ Developmental factors may well have played an essential role in handaxe manufacture. Acheulean toolmakers, for instance, would have inherited from other individuals niches containing stimuli related to handaxe production. Such niches would have provided cues for individual learning. Their layout would also have encouraged the task to be structured in very specific, previously tested ways.^118^

In accordance with much other work on cultural evolution, we have “black‐boxed” specific mechanisms driving the suggested genetic evolution of handaxes, such as stabilizing or directional selection, genetic drift, genetic assimilation, and Baldwinian evolution. However, like Sterelny^119^ and others,^120^ we see the Baldwin effect as a plausible candidate. Learned responses to environmental stimuli can be replaced by genetically controlled behaviors that occur with little or no learning as a result of selection pressure on the technical abilities of the species. Accumulation of technology‐related genes “steering” the developmental mechanism may thus gradually have resulted in a specific, adaptive, extended phenotype, with basic technological behaviors becoming innate.

Learned responses to environmental stimuli can be replaced by genetically controlled behaviors that occur with little or no learning as a result of selection pressure on the technical abilities of the species. Accumulation of technology‐related genes “steering” the developmental mechanism may thus gradually have resulted in a specific, adaptive, extended phenotype, with basic technological behaviors becoming instinctual.

Another issue that we have left open is the nature of the Oldowan^121^ and the recently discovered Lomekwian,^122^ the industries that preceded the Acheulean. It is tempting to argue that if the Acheulean handaxe was under genetic control, then the much simpler tool types of the Oldowan and Lomekian were probably also under genetic control. However, as we explained earlier, McElreath's^52^ finding that different mechanisms underpin different beliefs in his study groups means that there is no reason to expect that all tools in the same industry involved the same transmission mechanisms. Needless to say, assuming that three entire industries involved the same transmission mechanisms is even more problematic. Given this, we think the applicability of the genetic transmission hypothesis should be investigated in relation to pre‐Acheulean industries, but we are uncertain about what will be found. It is possible that stone‐tool technology went from being genetically controlled to purely cultural over the course of nearly three‐and‐a‐half million years. But it is also possible that stone‐tool technology went from purely cultural to genetically controlled to purely cultural again. Indeed, based on McElreath's^52^ results, we need to allow for an even more complicated scenario in which the three industries have different combinations of transmission mechanisms.

Finally it is worth noting that when nonhuman animals display complex behavior, the default assumption is that it is under genetic control. For complex behavior in humans and other hominins, however, the default position is to invoke culture and not genes. This double standard betrays a form of anthropocentrism. We contend that the genetic transmission hypothesis deserves serious consideration for this reason as well.^123^
